# Complex Problem Solving: What It Is and What It Is Not

**DOI:** 10.3389/fpsyg.2017.01153

**Published:** 2017-07-11

**Authors:** Dietrich Dörner, Joachim Funke

**Affiliations:** ^1^Department of Psychology, University of Bamberg Bamberg, Germany; ^2^Department of Psychology, Heidelberg University Heidelberg, Germany

**Keywords:** complex problem solving, validity, assessment, definition, MicroDYN

## Abstract

Computer-simulated scenarios have been part of psychological research on problem solving for more than 40 years. The shift in emphasis from simple toy problems to complex, more real-life oriented problems has been accompanied by discussions about the best ways to assess the process of solving complex problems. Psychometric issues such as reliable assessments and addressing correlations with other instruments have been in the foreground of these discussions and have left the content validity of complex problem solving in the background. In this paper, we return the focus to content issues and address the important features that define complex problems.

Succeeding in the 21st century requires many competencies, including creativity, life-long learning, and collaboration skills (e.g., National Research Council, 2011; Griffin and Care, 2015), to name only a few. One competence that seems to be of central importance is the ability to solve complex problems (Mainzer, 2009). Mainzer quotes the Nobel prize winner Simon (1957) who wrote as early as 1957:

The capacity of the human mind for formulating and solving complex problems is very small compared with the size of the problem whose solution is required for objectively rational behavior in the real world or even for a reasonable approximation to such objective rationality. (p. 198)

The shift from well-defined to ill-defined problems came about as a result of a disillusion with the “general problem solver” (Newell et al., 1959): The general problem solver was a computer software intended to solve all kind of problems that can be expressed through well-formed formulas. However, it soon became clear that this procedure was in fact a “special problem solver” that could only solve well-defined problems in a closed space. But real-world problems feature open boundaries and have no well-determined solution. In fact, the world is full of wicked problems and clumsy solutions (Verweij and Thompson, 2006). As a result, solving well-defined problems and solving ill-defined problems requires different cognitive processes (Schraw et al., 1995; but see Funke, 2010).

Well-defined problems have a clear set of means for reaching a precisely described goal state. For example: in a match-stick arithmetic problem, a person receives a false arithmetic expression constructed out of matchsticks (e.g., IV = III + III). According to the instructions, moving one of the matchsticks will make the equations true. Here, both the problem (find the appropriate stick to move) and the goal state (true arithmetic expression; solution is: VI = III + III) are defined clearly.

Ill-defined problems have no clear problem definition, their goal state is not defined clearly, and the means of moving towards the (diffusely described) goal state are not clear. For example: The goal state for solving the political conflict in the near-east conflict between Israel and Palestine is not clearly defined (living in peaceful harmony with each other?) and even if the conflict parties would agree on a two-state solution, this goal again leaves many issues unresolved. This type of problem is called a “complex problem” and is of central importance to this paper. All psychological processes that occur within individual persons and deal with the handling of such ill-defined complex problems will be subsumed under the umbrella term “complex problem solving” (CPS).

Systematic research on CPS started in the 1970s with observations of the behavior of participants who were confronted with computer simulated microworlds. For example, in one of those microworlds participants assumed the role of executives who were tasked to manage a company over a certain period of time (see Brehmer and Dörner, 1993, for a discussion of this methodology). Today, CPS is an established concept and has even influenced large-scale assessments such as PISA (“Programme for International Student Assessment”), organized by the Organization for Economic Cooperation and Development (OECD, 2014). According to the World Economic Forum, CPS is one of the most important competencies required in the future (World Economic Forum, 2015). Numerous articles on the subject have been published in recent years, documenting the increasing research activity relating to this field. In the following collection of papers we list only those published in 2010 and later: *theoretical* papers (Blech and Funke, 2010; Funke, 2010; Knauff and Wolf, 2010; Leutner et al., 2012; Selten et al., 2012; Wüstenberg et al., 2012; Greiff et al., 2013b; Fischer and Neubert, 2015; Schoppek and Fischer, 2015), papers about *measurement issues* (Danner et al., 2011a; Greiff et al., 2012, 2015a; Alison et al., 2013; Gobert et al., 2015; Greiff and Fischer, 2013; Herde et al., 2016; Stadler et al., 2016), papers about *applications* (Fischer and Neubert, 2015; Ederer et al., 2016; Tremblay et al., 2017), papers about *differential effects* (Barth and Funke, 2010; Danner et al., 2011b; Beckmann and Goode, 2014; Greiff and Neubert, 2014; Scherer et al., 2015; Meißner et al., 2016; Wüstenberg et al., 2016), one paper about *developmental effects* (Frischkorn et al., 2014), one paper with a *neuroscience* background (Osman, 2012)^1^, papers about *cultural differences* (Güss and Dörner, 2011; Sonnleitner et al., 2014; Güss et al., 2015), papers about *validity issues* (Goode and Beckmann, 2010; Greiff et al., 2013c; Schweizer et al., 2013; Mainert et al., 2015; Funke et al., 2017; Greiff et al., 2017, 2015b; Kretzschmar et al., 2016; Kretzschmar, 2017), *review papers* and *meta-analyses* (Osman, 2010; Stadler et al., 2015), and finally *books* (Qudrat-Ullah, 2015; Csapó and Funke, 2017b) and *book chapters* (Funke, 2012; Hotaling et al., 2015; Funke and Greiff, 2017; Greiff and Funke, 2017; Csapó and Funke, 2017a; Fischer et al., 2017; Molnàr et al., 2017; Tobinski and Fritz, 2017; Viehrig et al., 2017). In addition, a new “Journal of Dynamic Decision Making” (JDDM) has been launched (Fischer et al., 2015, 2016) to give the field an open-access outlet for research and discussion.

This paper aims to clarify aspects of validity: what should be meant by the term CPS and what not? This clarification seems necessary because misunderstandings in recent publications provide – from our point of view – a potentially misleading picture of the construct. We start this article with a historical review before attempting to systematize different positions. We conclude with a working definition.

## Historical Review

The concept behind CPS goes back to the German phrase “komplexes Problemlösen” (CPS; the term “komplexes Problemlösen” was used as a book title by Funke, 1986). The concept was introduced in Germany by Dörner and colleagues in the mid-1970s (see Dörner et al., 1975; Dörner, 1975) for the first time. The German phrase was later translated to CPS in the titles of two edited volumes by Sternberg and Frensch (1991) and Frensch and Funke (1995a) that collected papers from different research traditions. Even though it looks as though the term was coined in the 1970s, Edwards (1962) used the term “dynamic decision making” to describe decisions that come in a sequence. He compared static with dynamic decision making, writing:

In dynamic situations, a new complication not found in the static situations arises. The environment in which the decision is set may be changing, either as a function of the sequence of decisions, or independently of them, or both. It is this possibility of an environment which changes while you collect information about it which makes the task of dynamic decision theory so difficult and so much fun. (p. 60)

The ability to solve complex problems is typically measured via dynamic systems that contain several interrelated variables that participants need to alter. Early work (see, e.g., Dörner, 1980) used a simulation scenario called “Lohhausen” that contained more than 2000 variables that represented the activities of a small town: Participants had to take over the role of a mayor for a simulated period of 10 years. The simulation condensed these ten years to ten hours in real time. Later, researchers used smaller dynamic systems as scenarios either based on linear equations (see, e.g., Funke, 1993) or on finite state automata (see, e.g., Buchner and Funke, 1993). In these contexts, CPS consisted of the identification and control of dynamic task environments that were previously unknown to the participants. Different task environments came along with different degrees of fidelity (Gray, 2002).

According to Funke (2012), the typical attributes of complex systems are (a) *complexity* of the problem situation which is usually represented by the sheer number of involved variables; (b) *connectivity* and mutual dependencies between involved variables; (c) *dynamics* of the situation, which reflects the role of time and developments within a system; (d) *intransparency* (in part or full) about the involved variables and their current values; and (e) *polytely* (greek term for “many goals”), representing goal conflicts on different levels of analysis. This mixture of features is similar to what is called VUCA (volatility, uncertainty, complexity, ambiguity) in modern approaches to management (e.g., Mack et al., 2016).

In his evaluation of the CPS movement, Sternberg (1995) compared (young) European approaches to CPS with (older) American research on expertise. His analysis of the differences between the European and American traditions shows advantages but also potential drawbacks for each side. He states (p. 301): “I believe that although there are problems with the European approach, it deals with some fundamental questions that American research scarcely addresses.” So, even though the echo of the European approach did not enjoy strong resonance in the US at that time, it was valued by scholars like Sternberg and others. Before attending to validity issues, we will first present a short review of different streams.

## Different Approaches to CPS

In the short history of CPS research, different approaches can be identified (Buchner, 1995; Fischer et al., 2017). To systematize, we differentiate between the following five lines of research:

(a) The search for *individual differences* comprises studies identifying interindividual differences that affect the ability to solve complex problems. This line of research is reflected, for example, in the early work by Dörner et al. (1983) and their “Lohhausen” study. Here, naïve student participants took over the role of the mayor of a small simulated town named Lohhausen for a simulation period of ten years. According to the results of the authors, it is not intelligence (as measured by conventional IQ tests) that predicts performance, but it is the ability to stay calm in the face of a challenging situation and the ability to switch easily between an analytic mode of processing and a more holistic one.
(b) The search for *cognitive processes* deals with the processes behind understanding complex dynamic systems. Representative of this line of research is, for example, Berry and Broadbent’s (1984) work on implicit and explicit learning processes when people interact with a dynamic system called “Sugar Production”. They found that those who perform best in controlling a dynamic system can do so implicitly, without explicit knowledge of details regarding the systems’ relations.
(c) The search for *system factors* seeks to identify the aspects of dynamic systems that determine the difficulty of complex problems and make some problems harder than others. Representative of this line of research is, for example, work by Funke (1985), who systematically varied the number of causal effects within a dynamic system or the presence/absence of eigendynamics. He found, for example, that solution quality decreases as the number of systems relations increases.
(d) The *psychometric approach* develops measurement instruments that can be used as an alternative to classical IQ tests, as something that goes “beyond IQ”. The MicroDYN approach (Wüstenberg et al., 2012) is representative for this line of research that presents an alternative to reasoning tests (like Raven matrices). These authors demonstrated that a small improvement in predicting school grade point average beyond reasoning is possible with MicroDYN tests.
(e) The *experimental approach* explores CPS under different experimental conditions. This approach uses CPS assessment instruments to test hypotheses derived from psychological theories and is sometimes used in research about cognitive processes (see above). Exemplary for this line of research is the work by Rohe et al. (2016), who test the usefulness of “motto goals” in the context of complex problems compared to more traditional learning and performance goals. Motto goals differ from pure performance goals by activating positive affect and should lead to better goal attainment especially in complex situations (the mentioned study found no effect).

To be clear: these five approaches are not mutually exclusive and do overlap. But the differentiation helps to identify different research communities and different traditions. These communities had different opinions about scaling complexity.

## The Race for Complexity: Use of More and More Complex Systems

In the early years of CPS research, microworlds started with systems containing about 20 variables (“Tailorshop”), soon reached 60 variables (“Moro”), and culminated in systems with about 2000 variables (“Lohhausen”). This race for complexity ended with the introduction of the concept of “minimal complex systems” (MCS; Greiff and Funke, 2009; Funke and Greiff, 2017), which ushered in a search for the lower bound of complexity instead of the higher bound, which could not be defined as easily. The idea behind this concept was that whereas the upper limits of complexity are unbound, the lower limits might be identifiable. Imagine starting with a simple system containing two variables with a simple linear connection between them; then, step by step, increase the number of variables and/or the type of connections. One soon reaches a point where the system can no longer be considered simple and has become a “complex system”. This point represents a minimal complex system. Despite some research having been conducted in this direction, the point of transition from simple to complex has not been identified clearly as of yet.

Some years later, the original “minimal complex systems” approach (Greiff and Funke, 2009) shifted to the “multiple complex systems” approach (Greiff et al., 2013a). This shift is more than a slight change in wording: it is important because it taps into the issue of validity directly. *Minimal* complex systems have been introduced in the context of challenges from large-scale assessments like PISA 2012 that measure new aspects of problem solving, namely *interactive* problems besides static problem solving (Greiff and Funke, 2017). PISA 2012 required test developers to remain within testing time constraints (given by the school class schedule). Also, test developers needed a large item pool for the construction of a broad class of problem solving items. It was clear from the beginning that MCS deal with simple dynamic situations that require controlled interaction: the exploration and control of simple ticket machines, simple mobile phones, or simple MP3 players (all of these example domains were developed within PISA 2012) – rather than really *complex* situations like managerial or political decision making.

As a consequence of this subtle but important shift in interpreting the letters MCS, the definition of CPS became a subject of debate recently (Funke, 2014a; Greiff and Martin, 2014; Funke et al., 2017). In the words of Funke (2014b, p. 495):

It is funny that problems that nowadays come under the term ‘CPS’, are less complex (in terms of the previously described attributes of complex situations) than at the beginning of this new research tradition. The emphasis on psychometric qualities has led to a loss of variety. Systems thinking requires more than analyzing models with two or three linear equations – nonlinearity, cyclicity, rebound effects, etc. are inherent features of complex problems and should show up at least in some of the problems used for research and assessment purposes. Minimal complex systems run the danger of becoming minimal valid systems.

Searching for minimal complex systems is not the same as gaining insight into the way how humans deal with complexity and uncertainty. For psychometric purposes, it is appropriate to reduce complexity to a minimum; for understanding problem solving under conditions of overload, intransparency, and dynamics, it is necessary to realize those attributes with reasonable strength. This aspect is illustrated in the next section.

## Importance of the Validity Issue

The most important reason for discussing the question of what complex problem solving is and what it is not stems from its phenomenology: if we lose sight of our phenomena, we are no longer doing good psychology. The relevant phenomena in the context of complex problems encompass many important aspects. In this section, we discuss four phenomena that are specific to complex problems. We consider these phenomena as critical for theory development and for the construction of assessment instruments (i.e., microworlds). These phenomena require theories for explaining them and they require assessment instruments eliciting them in a reliable way.

The first phenomenon is the emergency reaction of the intellectual system (Dörner, 1980): When dealing with complex systems, actors tend to (a) reduce their intellectual level by decreasing self-reflections, by decreasing their intentions, by stereotyping, and by reducing their realization of intentions, (b) they show a tendency for fast action with increased readiness for risk, with increased violations of rules, and with increased tendency to escape the situation, and (c) they degenerate their hypotheses formation by construction of more global hypotheses and reduced tests of hypotheses, by increasing entrenchment, and by decontextualizing their goals. This phenomenon illustrates the strong *connection between cognition, emotion, and motivation* that has been emphasized by Dörner (see, e.g., Dörner and Güss, 2013) from the beginning of his research tradition; the emergency reaction reveals a shift in the mode of information processing under the pressure of complexity.

The second phenomenon comprises cross-cultural differences with respect to strategy use (Strohschneider and Güss, 1999; Güss and Wiley, 2007; Güss et al., 2015). Results from complex task environments illustrate the strong influence of *context and background knowledge* to an extent that cannot be found for knowledge-poor problems. For example, in a comparison between Brazilian and German participants, it turned out that Brazilians accept the given problem descriptions and are more optimistic about the results of their efforts, whereas Germans tend to inquire more about the background of the problems and take a more active approach but are less optimistic (according to Strohschneider and Güss, 1998, p. 695).

The third phenomenon relates to failures that occur during the planning and acting stages (Jansson, 1994; Ramnarayan et al., 1997), illustrating that rational procedures seem to be unlikely to be used in complex situations. The *potential for failures* (Dörner, 1996) rises with the complexity of the problem. Jansson (1994) presents seven major areas for failures with complex situations: acting directly on current feedback; insufficient systematization; insufficient control of hypotheses and strategies; lack of self-reflection; selective information gathering; selective decision making; and thematic vagabonding.

The fourth phenomenon describes (a lack of) training and transfer effects (Kretzschmar and Süß, 2015), which again illustrates the *context dependency of strategies and knowledge* (i.e., there is no strategy that is so universal that it can be used in many different problem situations). In their own experiment, the authors could show training effects only for knowledge acquisition, not for knowledge application. Only with specific feedback, performance in complex environments can be increased (Engelhart et al., 2017).

These four phenomena illustrate why the type of complexity (or degree of simplicity) used in research really matters. Furthermore, they demonstrate effects that are specific for complex problems, but not for toy problems. These phenomena direct the attention to the important question: does the stimulus material used (i.e., the computer-simulated microworld) tap and elicit the manifold of phenomena described above?

Dealing with partly unknown complex systems requires courage, wisdom, knowledge, grit, and creativity. In creativity research, “little c” and “BIG C” are used to differentiate between everyday creativity and eminent creativity (Beghetto and Kaufman, 2007; Kaufman and Beghetto, 2009). Everyday creativity is important for solving everyday problems (e.g., finding a clever fix for a broken spoke on my bicycle), eminent creativity changes the world (e.g., inventing solar cells for energy production). Maybe problem solving research should use a similar differentiation between “little p” and “BIG P” to mark toy problems on the one side and big societal challenges on the other. The question then remains: what can we learn about BIG P by studying little p? What phenomena are present in both types, and what phenomena are unique to each of the two extremes?

## On Methods

Discussing research on CPS requires reflecting on the field’s research methods. Even if the experimental approach has been successful for testing hypotheses (for an overview of older work, see Funke, 1995), other methods might provide additional and novel insights. Complex phenomena require complex approaches to understand them. The complex nature of complex systems imposes limitations on psychological experiments: The more complex the environments, the more difficult is it to keep conditions under experimental control. And if experiments have to be run in labs one should bring enough complexity into the lab to establish the phenomena mentioned, at least in part.

There are interesting options to be explored (again): *think-aloud protocols*, which have been discredited for many years (Nisbett and Wilson, 1977) and yet are a valuable source for theory testing (Ericsson and Simon, 1983); *introspection* (Jäkel and Schreiber, 2013), which seems to be banned from psychological methods but nevertheless offers insights into thought processes; the use of *life-streaming* (Wendt, 2017), a medium in which streamers generate a video stream of think-aloud data in computer-gaming; *political decision-making* (Dhami et al., 2015) that demonstrates error-proneness in groups; historical *case studies* (Dörner and Güss, 2011) that give insights into the thinking styles of political leaders; the use of the *critical incident* technique (Reuschenbach, 2008) to construct complex scenarios; and simulations with different degrees of *fidelity* (Gray, 2002).

The methods tool box is full of instruments that have to be explored more carefully before any individual instrument receives a ban or research narrows its focus to only one paradigm for data collection. Brehmer and Dörner (1993) discussed the tensions between “research in the laboratory and research in the field”, optimistically concluding “that the new methodology of computer-simulated microworlds will provide us with the means to bridge the gap between the laboratory and the field” (p. 183). The idea behind this optimism was that computer-simulated scenarios would bring more complexity from the outside world into the controlled lab environment. But this is not true for all simulated scenarios. In his paper on simulated environments, Gray (2002) differentiated computer-simulated environments with respect to three dimensions: (1) tractability (“the more training subjects require before they can use a simulated task environment, the less tractable it is”, p. 211), correspondence (“High correspondence simulated task environments simulate many aspects of one task environment. Low correspondence simulated task environments simulate one aspect of many task environments”, p. 214), and engagement (“A simulated task environment is engaging to the degree to which it involves and occupies the participants; that is, the degree to which they agree to take it seriously”, p. 217). But the mere fact that a task is called a “computer-simulated task environment” does not mean anything specific in terms of these three dimensions. This is one of several reasons why we should differentiate between those studies that do not address the core features of CPS and those that do.

## What is not CPS?

Even though a growing number of references claiming to deal with complex problems exist (e.g., Greiff and Wüstenberg, 2015; Greiff et al., 2016), it would be better to label the requirements within these tasks “dynamic problem solving,” as it has been done adequately in earlier work (Greiff et al., 2012). The dynamics behind on-off-switches (Thimbleby, 2007) are remarkable but not really complex. Small nonlinear systems that exhibit stunningly complex and unstable behavior do exist – but they are not used in psychometric assessments of so-called CPS. There are other small systems (like MicroDYN scenarios: Greiff and Wüstenberg, 2014) that exhibit simple forms of system behavior that are completely predictable and stable. This type of simple systems is used frequently. It is even offered commercially as a complex problem-solving test called COMPRO (Greiff and Wüstenberg, 2015) for business applications. But a closer look reveals that the label is not used correctly; within COMPRO, the used linear equations are far from being complex and the system can be handled properly by using only one strategy (see for more details Funke et al., 2017).

Why do simple linear systems not fall within CPS? At the surface, nonlinear and linear systems might appear similar because both only include 3–5 variables. But the difference is in terms of systems behavior as well as strategies and learning. If the behavior is simple (as in linear systems where more input is related to more output and vice versa), the system can be easily understood (participants in the MicroDYN world have 3 minutes to explore a complex system). If the behavior is complex (as in systems that contain strange attractors or negative feedback loops), things become more complicated and much more observation is needed to identify the hidden structure of the unknown system (Berry and Broadbent, 1984; Hundertmark et al., 2015).

Another issue is learning. If tasks can be solved using a single (and not so complicated) strategy, steep learning curves are to be expected. The shift from problem solving to learned routine behavior occurs rapidly, as was demonstrated by Luchins (1942). In his water jar experiments, participants quickly acquired a specific strategy (a mental set) for solving certain measurement problems that they later continued applying to problems that would have allowed for easier approaches. In the case of complex systems, learning can occur only on very general, abstract levels because it is difficult for human observers to make specific predictions. Routines dealing with complex systems are quite different from routines relating to linear systems.

What should not be studied under the label of CPS are pure learning effects, multiple-cue probability learning, or tasks that can be solved using a single strategy. This last issue is a problem for MicroDYN tasks that rely strongly on the VOTAT strategy (“vary one thing at a time”; see Tschirgi, 1980). In real-life, it is hard to imagine a business manager trying to solve her or his problems by means of VOTAT.

## What is CPS?

In the early days of CPS research, planet Earth’s dynamics and complexities gained attention through such books as “The limits to growth” (Meadows et al., 1972) and “Beyond the limits” (Meadows et al., 1992). In the current decade, for example, the World Economic Forum (2016) attempts to identify the complexities and risks of our modern world. In order to understand the meaning of complexity and uncertainty, taking a look at the worlds’ most pressing issues is helpful. Searching for strategies to cope with these problems is a difficult task: surely there is no place for the simple principle of “vary-one-thing-at-a-time” (VOTAT) when it comes to global problems. The VOTAT strategy is helpful in the context of simple problems (Wüstenberg et al., 2014); therefore, whether or not VOTAT is helpful in a given problem situation helps us distinguish simple from complex problems.

Because there exist no clear-cut strategies for complex problems, typical failures occur when dealing with uncertainty (Dörner, 1996; Güss et al., 2015). Ramnarayan et al. (1997) put together a list of generic errors (e.g., not developing adequate action plans; lack of background control; learning from experience blocked by stereotype knowledge; reactive instead of proactive action) that are typical of knowledge-rich complex systems but cannot be found in simple problems.

Complex problem solving is not a one-dimensional, low-level construct. On the contrary, CPS is a multi-dimensional bundle of competencies existing at a high level of abstraction, similar to intelligence (but going beyond IQ). As Funke et al. (2018) state: “Assessment of transversal (in educational contexts: cross-curricular) competencies cannot be done with one or two types of assessment. The plurality of skills and competencies requires a plurality of assessment instruments.”

There are at least three different aspects of complex systems that are part of our understanding of a complex system: (1) a complex system can be described at different levels of abstraction; (2) a complex system develops over time, has a history, a current state, and a (potentially unpredictable) future; (3) a complex system is knowledge-rich and activates a large semantic network, together with a broad list of potential strategies (domain-specific as well as domain-general).

Complex problem solving is not only a cognitive process but is also an emotional one (Spering et al., 2005; Barth and Funke, 2010) and strongly dependent on motivation (low-stakes versus high-stakes testing; see Hermes and Stelling, 2016).

Furthermore, CPS is a dynamic process unfolding over time, with different phases and with more differentiation than simply knowledge acquisition and knowledge application. Ideally, the process should entail identifying problems (see Dillon, 1982; Lee and Cho, 2007), even if in experimental settings, problems are provided to participants *a priori*. The more complex and open a given situation, the more options can be generated (T. S. Schweizer et al., 2016). In closed problems, these processes do not occur in the same way.

In analogy to the difference between formative (process-oriented) and summative (result-oriented) assessment (Wiliam and Black, 1996; Bennett, 2011), CPS should not be reduced to the mere outcome of a solution process. The process leading up to the solution, including detours and errors made along the way, might provide a more differentiated impression of a person’s problem-solving abilities and competencies than the final result of such a process. This is one of the reasons why CPS environments are not, in fact, complex intelligence tests: research on CPS is not only about the outcome of the decision process, but it is also about the problem-solving process itself.

Complex problem solving is part of our daily life: finding the right person to share one’s life with, choosing a career that not only makes money, but that also makes us happy. Of course, CPS is not restricted to personal problems – life on Earth gives us many hard nuts to crack: climate change, population growth, the threat of war, the use and distribution of natural resources. In sum, many societal challenges can be seen as complex problems. To reduce that complexity to a one-hour lab activity on a random Friday afternoon puts it out of context and does not address CPS issues.

Theories about CPS should specify which populations they apply to. Across populations, one thing to consider is prior knowledge. CPS research with experts (e.g., Dew et al., 2009) is quite different from problem solving research using tasks that intentionally do not require any specific prior knowledge (see, e.g., Beckmann and Goode, 2014).

More than 20 years ago, Frensch and Funke (1995b) defined CPS as follows:

CPS occurs to overcome barriers between a given state and a desired goal state by means of behavioral and/or cognitive, multi-step activities. The given state, goal state, and barriers between given state and goal state are complex, change dynamically during problem solving, and are intransparent. The exact properties of the given state, goal state, and barriers are unknown to the solver at the outset. CPS implies the efficient interaction between a solver and the situational requirements of the task, and involves a solver’s cognitive, emotional, personal, and social abilities and knowledge. (p. 18)

The above definition is rather formal and does not account for content or relations between the simulation and the real world. In a sense, we need a new definition of CPS that addresses these issues. Based on our previous arguments, we propose the following working definition:

Complex problem solving is a collection of self-regulated psychological processes and activities necessary in dynamic environments to achieve ill-defined goals that cannot be reached by routine actions. Creative combinations of knowledge and a broad set of strategies are needed. Solutions are often more bricolage than perfect or optimal. The problem-solving process combines cognitive, emotional, and motivational aspects, particularly in high-stakes situations. Complex problems usually involve knowledge-rich requirements and collaboration among different persons.

The main differences to the older definition lie in the emphasis on (a) the self-regulation of processes, (b) creativity (as opposed to routine behavior), (c) the bricolage type of solution, and (d) the role of high-stakes challenges. Our new definition incorporates some aspects that have been discussed in this review but were not reflected in the 1995 definition, which focused on attributes of complex problems like dynamics or intransparency.

This leads us to the final reflection about the role of CPS for dealing with uncertainty and complexity in real life. We will distinguish thinking from reasoning and introduce the sense of possibility as an important aspect of validity.

## CPS as Combining Reasoning and Thinking in an Uncertain Reality

Leading up to the Battle of Borodino in Leo Tolstoy’s novel “War and Peace”, Prince Andrei Bolkonsky explains the concept of war to his friend Pierre. Pierre expects war to resemble a game of chess: You position the troops and attempt to defeat your opponent by moving them in different directions.

“Far from it!”, Andrei responds. “In chess, you know the knight and his moves, you know the pawn and his combat strength. While in war, a battalion is sometimes stronger than a division and sometimes weaker than a company; it all depends on circumstances that can never be known. In war, you do not know the position of your enemy; some things you might be able to observe, some things you have to divine (but that depends on your ability to do so!) and many things cannot even be guessed at. In chess, you can see all of your opponent’s possible moves. In war, that is impossible. If you decide to attack, you cannot know whether the necessary conditions are met for you to succeed. Many a time, you cannot even know whether your troops will follow your orders…”

In essence, war is characterized by a high degree of uncertainty. A good commander (or politician) can add to that what he or she sees, tentatively fill in the blanks – and not just by means of logical deduction but also by intelligently bridging missing links. A bad commander extrapolates from what he sees and thus arrives at improper conclusions.

Many languages differentiate between two modes of mentalizing; for instance, the English language distinguishes between ‘thinking’ and ‘reasoning’. Reasoning denotes acute and exact mentalizing involving logical deductions. Such deductions are usually based on evidence and counterevidence. Thinking, however, is what is required to write novels. It is the construction of an initially unknown reality. But it is not a pipe dream, an unfounded process of fabrication. Rather, thinking asks us to imagine reality (“Wirklichkeitsfantasie”). In other words, a novelist has to possess a “sense of possibility” (“Möglichkeitssinn”, Robert Musil; in German, sense of possibility is often used synonymously with imagination even though imagination is not the same as sense of possibility, for imagination also encapsulates the impossible). This sense of possibility entails knowing the whole (or several wholes) or being able to construe an unknown whole that could accommodate a known part. The whole has to align with sociological and geographical givens, with the mentality of certain peoples or groups, and with the laws of physics and chemistry. Otherwise, the entire venture is ill-founded. A sense of possibility does not aim for the moon but imagines something that might be possible but has not been considered possible or even potentially possible so far.

Thinking is a means to eliminate uncertainty. This process requires both of the modes of thinking we have discussed thus far. Economic, political, or ecological decisions require us to first consider the situation at hand. Though certain situational aspects can be known, but many cannot. In fact, von Clausewitz (1832) posits that only about 25% of the necessary information is available when a military decision needs to be made. Even then, there is no way to guarantee that whatever information is available is also correct: Even if a piece of information was completely accurate yesterday, it might no longer apply today.

Once our sense of possibility has helped grasping a situation, problem solvers need to call on their reasoning skills. Not every situation requires the same action, and we may want to act this way or another to reach this or that goal. This appears logical, but it is a logic based on constantly shifting grounds: We cannot know whether necessary conditions are met, sometimes the assumptions we have made later turn out to be incorrect, and sometimes we have to revise our assumptions or make completely new ones. It is necessary to constantly switch between our sense of possibility and our sense of reality, that is, to switch between thinking and reasoning. It is an arduous process, and some people handle it well, while others do not.

If we are to believe Tuchman’s (1984) book, “The March of Folly”, most politicians and commanders are fools. According to Tuchman, not much has changed in the 3300 years that have elapsed since the misguided Trojans decided to welcome the left-behind wooden horse into their city that would end up dismantling Troy’s defensive walls. The Trojans, too, had been warned, but decided not to heed the warning. Although Laocoön had revealed the horse’s true nature to them by attacking it with a spear, making the weapons inside the horse ring, the Trojans refused to see the forest for the trees. They did not want to listen, they wanted the war to be over, and this desire ended up shaping their perception.

The objective of psychology is to predict and explain human actions and behavior as accurately as possible. However, thinking cannot be investigated by limiting its study to neatly confined fractions of reality such as the realms of propositional logic, chess, Go tasks, the Tower of Hanoi, and so forth. Within these systems, there is little need for a sense of possibility. But a sense of possibility – the ability to divine and construe an unknown reality – is at least as important as logical reasoning skills. Not researching the sense of possibility limits the validity of psychological research. All economic and political decision making draws upon this sense of possibility. By not exploring it, psychological research dedicated to the study of thinking cannot further the understanding of politicians’ competence and the reasons that underlie political mistakes. Christopher Clark identifies European diplomats’, politicians’, and commanders’ inability to form an accurate representation of reality as a reason for the outbreak of World War I. According to Clark’s (2012) book, “The Sleepwalkers”, the politicians of the time lived in their own make-believe world, wrongfully assuming that it was the same world everyone else inhabited. If CPS research wants to make significant contributions to the world, it has to acknowledge complexity and uncertainty as important aspects of it.

## Conclusion

For more than 40 years, CPS has been a new subject of psychological research. During this time period, the initial emphasis on analyzing how humans deal with complex, dynamic, and uncertain situations has been lost. What is subsumed under the heading of CPS in modern research has lost the original complexities of real-life problems. From our point of view, the challenges of the 21st century require a return to the origins of this research tradition. We would encourage researchers in the field of problem solving to come back to the original ideas. There is enough complexity and uncertainty in the world to be studied. Improving our understanding of how humans deal with these global and pressing problems would be a worthwhile enterprise.

## Author Contributions

JF drafted a first version of the manuscript, DD added further text and commented on the draft. JF finalized the manuscript.

## Authors Note

After more than 40 years of controversial discussions between both authors, this is the first joint paper. We are happy to have done this now! We have found common ground!

## Conflict of Interest Statement

The authors declare that the research was conducted in the absence of any commercial or financial relationships that could be construed as a potential conflict of interest.

## Footnotes

1 The fMRI-paper from Anderson (2012) uses the term “complex problem solving” for tasks that do not fall in our understanding of CPS and is therefore excluded from this list.
